# Uterine Repair Mechanisms Are Potentiated by Mesenchymal Stem Cells and Decellularized Tissue Grafts Through Elevated *Vegf*, *Cd44*, and *Itgb1* Gene Expression

**DOI:** 10.3390/bioengineering11121268

**Published:** 2024-12-14

**Authors:** Sara Bandstein, Lucia De Miguel-Gómez, Edina Sehic, Emy Thorén, Sara López-Martínez, Irene Cervelló, Randa Akouri, Mihai Oltean, Mats Brännström, Mats Hellström

**Affiliations:** 1 Laboratory for Transplantation and Regenerative Medicine, Sahlgrenska Academy, University of Gothenburg, SE-405 30 Gothenburg, Sweden; sara.bandstein@gmail.com (S.B.); lucia.de.miguel.gomez@gu.se (L.D.M.-G.); edina.sehic@uu.se (E.S.); emy.thoren@gu.se (E.T.); randaakouri@hotmail.com (R.A.); mihai.oltean@surgery.gu.se (M.O.); mats.brannstrom@obgyn.gu.se (M.B.); 2 Department of Obstetrics and Gynecology, Clinical Sciences, Sahlgrenska Academy, University of Gothenburg, SE-405 30 Gothenburg, Sweden; 3 IVIRMA Global Research Alliance, IVI Foundation, Instituto de Investigación Sanitaria La Fe (IIS La Fe), Avenida Fernando Abril Martorell, 106, 46026 Valencia, Spain; saralopezmartinez94@hotmail.com (S.L.-M.); irene.cervello@ivirma.com (I.C.); 4 Department of Surgery, Clinical Sciences, Sahlgrenska Academy, University of Gothenburg, SE-413 45 Gothenburg, Sweden; 5 Stockholm IVF-EUGIN, Hammarby allé 93, SE-120 63 Stockholm, Sweden; 6 Health Innovation Labs by Sahlgrenska Science Park, Medicinaregatan 9C, SE-413 90 Gothenburg, Sweden

**Keywords:** uterus factor infertility, bioengineering, decellularized scaffolds, mesenchymal stem cells, infertility, uterus transplantation

## Abstract

Transplantation of decellularized uterus tissue showed promise in supporting regeneration following uterine injury in animal models, suggesting an alternative to complete uterus transplantation for uterine factor infertility treatment. However, most animal studies utilized small grafts, limiting their clinical relevance. Hence, we used larger grafts (20 × 10 mm), equivalent to nearly one uterine horn in rats, to better evaluate the bioengineering challenges associated with structural support, revascularization, and tissue regeneration. We analyzed histopathology, employed immunohistochemistry, and investigated gene expression discrepancies in growth-related proteins over four months post-transplantation in acellular grafts and those recellularized (RC) with bone marrow-derived mesenchymal stem cells (bmMSCs). RC grafts exhibited less inflammation and faster epithelialization and migration of endogenous cells into the graft compared with acellular grafts. Despite the lack of a significant difference in the density of CD31 positive blood vessels between groups, the RC group demonstrated a better organized myometrial layer and an overall faster regenerative progress. Elevated gene expression for *Vegf*, *Cd44*, and *Itgb1* correlated with the enhanced tissue regeneration in this group. Elevated *Tgfb* expression was noted in both groups, potentially contributing to the rapid revascularization. Our findings suggest that large uterine injuries can be regenerated using decellularized tissue, with bmMSCs enhancing the endogenous repair mechanisms.

## 1. Introduction

Bioengineering applications have extensively explored the development of personalized donor tissues using biomaterials and cells for transplantation across various organs and animal models. One common methodology includes the creation of tissue-specific scaffolds through a process known as decellularization. This approach involves the removal of most immunogenic cellular components from a donor organ while preserving the extracellular matrix (ECM) in a tissue-specific manner. Additionally, the preservation of patent vascular conduits would enable scaffold implantation via true transplantation, complete with vascular anastomoses and immediate blood supply. Furthermore, some decellularization protocols retain the scaffold’s bioactivity, stimulating angiogenic and tissue regeneration pathways, even after exposure to harsh detergents and other chemicals used in the protocols [1].

Due to these advantages, decellularized scaffolds have been extensively examined for application in reproductive bioengineering, particularly for treating infertility caused by uterine disorders [2]. Many studies have utilized a bioengineered uterus patch transplantation model that mimics clinical scenarios where it could be relevant to cure uterine factor infertility by replacing uterine scars or defects which may have occurred after clinical interventions such as myomectomy, adenomyomectomy, resection of placental tumors, or uterine rupture [3]. Collective results have demonstrated that decellularized tissue scaffolds stimulate regeneration and restore fertility in rodent models [4,5,6,7]. With the ambition to translate these promising studies into clinical applications, numerous research groups have developed scaffolds from decellularized uterine tissue in various species, including mice [8], rats [5,6,7], rabbits [9,10], cats [11], pigs [12], sheep [1,13], goats [14], cows [15], baboons [16], and humans [17,18].

A major challenge in using decellularized tissue as a biological scaffold is to achieve effective recellularization prior to engraftment. Nevertheless, even a sparsely repopulated scaffold with bone marrow-derived mesenchymal stem cells (bmMSCs) has shown advantageous regenerative outcomes in a rat model [19]. In the latter study, bmMSCs provided an immune-privileged microenvironment and shifted the endogenous leukocyte response to a regenerative state, thereby reducing the negative effects of damage-associated molecular patterns (DAMPs), which are generated during the decellularization process [20].

The first in vivo large animal model, utilizing the sheep, confirmed these bioengineering principles where a 6 cm^2^ bioengineered uterus patch, similar in size to what may be required in a human setting, was used. However, the short-term study results (six weeks) showed inconsistency in regeneration between the grafts within specific study groups, possibly due to differences in T-cell response [21]. However, other factors may also be involved, which mandates further studies. Therefore, continued rodent studies are important to further investigate the regeneration scheme over a longer timeframe, as they are less complex and less resource-demanding compared to large animal studies.

Hence, we implemented a 20 × 10 mm bioengineered uterus graft derived from decellularized uterus tissue, which in size is comparable to a full uterus horn in the rat model. After transplantation, we thoroughly analyzed the regeneration and histopathological events over a period of up to four months post-transplantation and conducted additional gene expression analysis to map specific repair mechanisms that could be potentiated by added bmMSCs.

## 2. Methods

### 2.1. Animals and Study Groups

**Figure 1:**
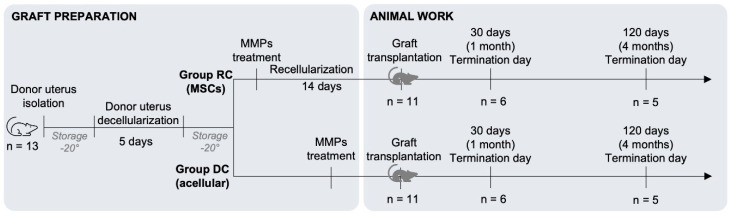
Experimental design. MMP, metalloproteinases; bmMSCs, bone marrow derived mesenchymal stem cells.

Forty-one female Sprague Dawley (SD) rats, aged 10–12 weeks, were obtained from Charles River (Sulzfeld, Germany). Thirteen rats served as donors for generating uterus scaffolds. Twenty-two rats were randomly assigned as recipients, where eleven received decellularized uterine tissue which had been recellularized in vitro with bmMSCs (Group RC), and eleven received decellularized uterine tissue without cells (Group DC). The grafts were assessed at 30 days (n = 6 per group) and 120 days post-transplantation (n = 5 per group; Figure 1). Additionally, six unmanipulated rats of the same age and breed were used to harvest normal tissue, serving as controls (Group Native). All animal procedures were approved by the local animal ethics committee in Gothenburg, Sweden, prior to the commencement of the study (document 2228/2019).

### 2.2. Uterus Isolation

Donor animals were deeply anesthetized with isoflurane (2–3%), and the uterus was explanted as previously described in detail [7]. Briefly, the organ was isolated, keeping the vascular trees to the uterus, via uterine, internal iliac, and common iliac intact. All other vascular branches were ligated and cut. The isolated organ was then perfused on the back table with a solution of xylocaine (vasodilating agent; 0.4 mg/mL, AstraZeneca, Gothenburg, Sweden) and heparinized phosphate-buffered saline (PBS; 50 IU/mL, Leo Pharma, Copenhagen, Denmark) by cannulating the aorta, leaving the vena cava outflow open. Once all blood was washed out, the donor uterus was stored at −20 °C in the perfusion solution until thawed for the immediate start of the decellularization process.

### 2.3. Decellularization by Vascular Perfusion

We employed a decellularization protocol extensively optimized and examined in previous studies [4,20]. Briefly, the organ was perfused at room temperature (RT) using a Masterflex perfusion pump (Cole-Parmer instruments, Chicago, IL, USA) that maintained a constant flow rate of 6 mL/min with a vascular pressure below 80 mm Hg throughout the process (pressure meter from Hugo Sachs Electronic-Harvard Apparatus GmbH; March-Hugstetten, Germany). Dimethyl sulfoxide (DMSO, 4% in deionized water; Sigma-Aldrich, Gothenburg, Sweden) was first perfused for 4 h, followed by Triton X-100 (1% in deionized water; Sigma-Aldrich) for another 4 h. Perfusion was then continued with deionized water overnight (16 h). This 24 h cycle was repeated for a total of five days. The decellularized uterus was then sterilized by perfusing peracetic acid (0.1%; purity 38–40%, Merck KGaA, Darmstadt, Germany) through the specimen’s vascular conduits for 1 h. Traces of peracetic acid were then removed by several washes with sterile PBS. The decellularized uteri were then frozen again in sterile PBS supplemented with 10% DMSO (Sigma-Aldrich) as a cryoprotectant until further use.

### 2.4. Recellularization and Graft Preparation

Each decellularized uterus was slowly thawed at 4 °C overnight, and the surrounding adipose tissue and vasculature were carefully excised. Each uterine horn was then longitudinally opened and trimmed to produce scaffold pieces measuring 20 mm × 10 mm. All scaffolds (n = 22) underwent enzymatic treatment to enhance porosity and improve recellularization efficiency, adhering to a previously established preconditioning protocol [1,19]. Specifically, matrix metalloproteinase (MMP) 2 (2.5 μg/L, Peprotech, Stockholm, Sweden) and MMP 9 (2.5 μg/L, Sigma-Aldrich, St. Louis, MO, USA) were prepared using an amino-phenylmercuric acetate buffer according to the manufacturer’s guidelines. Each scaffold was fully immersed and incubated for 24 h at 37 °C in the MMP2/9 solution. Following enzyme treatment, the scaffolds were washed twice in 20 mM EDTA (Sigma-Aldrich) for 20 min each, followed by two washes in sterile PBS for a total of 20 min. Eleven acellular grafts were placed in Leibovitz L-15 medium (Merck) and maintained at 37 °C for up to six hours before transplantation. The remaining 11 scaffolds were recellularized with 20 million OriCell^TM^ SD rat bone marrow-derived green fluorescent protein (GFP)-labeled MSCs (passage number < 7; Cyagen Biosciences, cat no. RASMX-01101, Santa Clara, CA, USA) via multiple injections using a 30G needle and 1 mL syringe. Briefly, the cells were resuspended in 1.2 mL of cell culture medium (DMEM GlutaMAX supplemented with 10% FBS and 1% Anti-Anti, Thermo Fisher Scientific, Waltham, Massachusetts, USA). Half of the total volume (0.6 mL) was then injected into the luminal side of the patch, and the rest of the cells were injected into the perimetrial side of the patch using 10–12 injections per patch side. The recellularized scaffolds were placed at 37 °C in trans-well cell culture inserts in 6-well plates (Sarstedt, Nümbrecht, Germany). After 3 h, the scaffolds were submerged in cell culture medium and incubated individually for two weeks under standard cell culture conditions. The culture medium was refreshed every second day.

### 2.5. Transplantation of Bioengineered Uterus Construct

Under 2–3% isoflurane anesthesia, a laparotomy was performed on each rat to expose the left uterine horn. A 20 × 10 mm full-thickness segment of the antemesometrial uterine wall was excised, leaving the recipient’s large blood vessel-containing uterine mesometrium intact. The excised segment was replaced with either an enzyme-treated recellularized scaffold (n = 11; Group RC) or an enzyme-treated acellular scaffold (n = 11; Group DC). The grafts were sutured in place using 6–0 non-absorbable polypropylene (Ethicon, Raritan, NJ, USA) with continuous sutures along the mesodermal side and interrupted sutures at the proximal and distal anastomosis sites. The abdominal muscle layer was closed with 4–0 silk sutures (Ethicon), and the skin was secured with titanium clips (Reflex7, Gaithersburg, MD, USA). Postoperative care included the administration of analgesics and antibiotics (buprenorphine 0.05 mg/kg; carprofen 5 mg/kg; sulfamethoxazole 100 mg/kg; trimethoprim 20 mg/kg). The transplanted scaffolds in Groups RC and DC were evaluated at 30 days (n = 6 per group) and 120 days (n = 5 per group) post-transplantation.

### 2.6. Staining and Immunohistochemistry

Five-micron thick sections of formalin-fixed, paraffin-embedded biopsies from grafted tissue were cut and mounted on slides. Hematoxylin and eosin staining (H&E; Histolab, Mölndal, Sweden) and Masson’s trichrome staining (MT; HT15-1KT, Sigma-Aldrich) were conducted according to standard protocols. For antibody-based protocols, a heat-induced antigen retrieval step was first performed on deparaffined and further rehydrated sections, using sodium citrate buffer (pH = 6) with 0.05% Tween 20 in a pressure cooker on full pressure for three minutes. Primary antibodies against E-cadherin (mouse monoclonal antibody ab76055; 1:250, Abcam, Cambridge, UK) and smooth-muscle cell actin (αSMA, rabbit recombinant monoclonal ab32575; 1:500, Abcam) were used for fluorescent immunohistochemistry by incubation overnight at 4 °C followed by a secondary fluorescent antibody (goat anti-mouse IgG Alexa Fluor^®^ 594, Ab150080; 1:300, Abcam and goat anti-rabbit IgG Alexa Fluor^®^ 498, Ab150077, 1:300, Abcam) for 1 h at RT. Nuclei were labeled using 4′,6-diamidino-2-phenylindole (DAPI, Sigma-Aldrich). A primary antibody against the endothelial-specific marker CD31 (Ab182981; 1:500, Abcam) was also used, but was visualized using a biotin-free alkaline phosphatase MACH 3™ polymer detection kit and the subsequent Vulcan Fast Red™ kit according to the manufacturer’s instructions (Biocare Medical, Pacheco, CA, USA). A negative control slide, with lack of primary antibody, was always included.

### 2.7. Tissue Regeneration and Vascular Density Assessment

**Figure 2:**
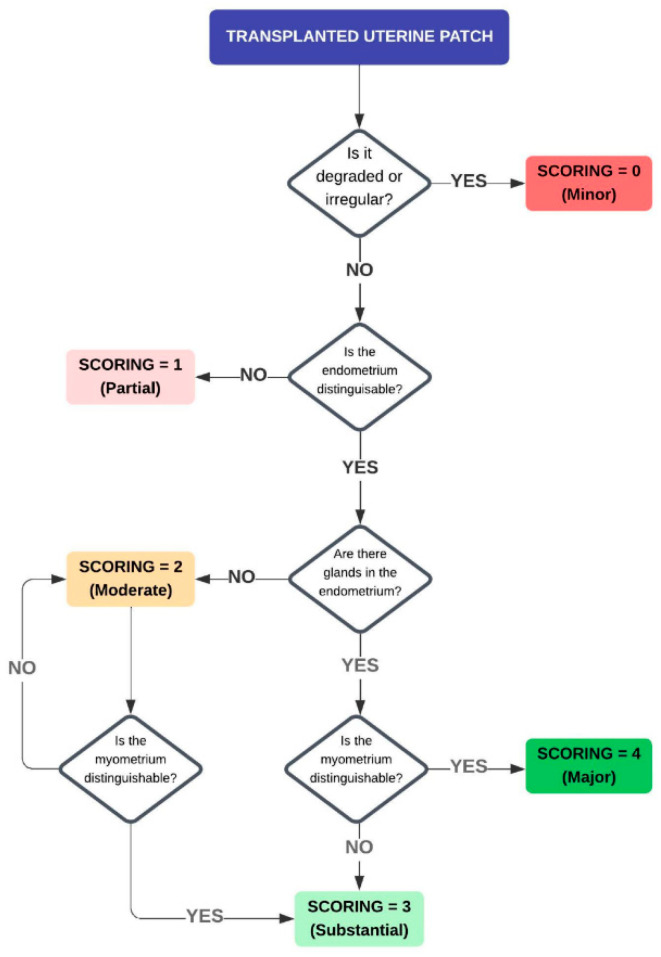
Decision diagram for the evaluation of uterine tissue regeneration. Created with Lucidchart software.

The general morphology was assessed using light microscopy after H&E and MT staining by two investigators blinded to the study groups. Multiple sections of each graft were independently scored using a 5-grade scoring system, adopted from an earlier publication assessing uterus tissue regeneration in the sheep [21,22] as detailed in Figure 2 (Figure 2). In brief, the regenerative outcomes were categorized as Minor (0), Partial (1), Moderate (2), Substantial (3), and Major (4) following one and four months after transplantation.

Additionally, the uterine thickness was measured from two cross-sections of each uterus graft using QuPath, and the total area was averaged and then compared to cross-sectioned normal uterus tissue [23].

Endometrial glandular tissue was quantified by counting the number of glands in eight randomly selected fields of the endometrium in the grafts from the two different time points. The value was then calculated and presented as the number of glands per mm^2^ and compared with control normal uterus tissue.

Finally, CD31^+^ endothelial cells enabled the identification of blood vessels. Hence, the number of blood vessels were quantified by two different observers blinded to the study groups in six random areas in sections from grafts and normal uterus tissue, respectively (200× magnification; 0.02 mm^2^/field).

### 2.8. Gene Expression Analysis: Digital Droplet PCR

Total RNA was extracted from RNAlater^TM^-preserved biopsies or from formalin-fixed, paraffin-embedded tissue sections (native uterine tissue, Group RC and Group DC) using the RNeasy FFPE kit (ref. 73504; Qiagen, Hilden, Germany). The RNA concentration and purity (ratios A260/280 and A260/230) values were evaluated using a NanoDrop spectrophotometer. The iScript cDNA synthesis kit (BioRad, Stockholm, Sweden) was used for reverse transcription. Briefly, 2 μL of each RNA sample, 20 μL of the manufacturer’s provided master mix, and 18 μL of nuclease-free water were mixed and subjected to cDNA conversion. cDNA samples were then droplet generated using a QX200™ droplet generator (BioRad). The droplet-partitioned samples were then amplified in a C1000 Touch Thermal Cycler (BioRad) and the fluorescence was measured using a QX2000 droplet reader (BioRad). Data were analyzed using the QuantaSoft™ software (version 1.7.4, BioRad). The number of copies/μL was normalized with the reference gene (relative gene expression) before transforming data to fold change (FC). FC was calculated by dividing the relative gene expression of every experimental group by the average gene expression value of the native uterine tissue (control group). FC was plotted in the graphs as Log_2_(FC). All steps followed the MIQE guidelines for ddPCR [24]. Pre-designed hydrolysis probes were obtained from BioRad, and target genes included cluster of differentiation 44 (*Cd44*; dRnoCPE5148382), chemokine (C-X-C motif) ligand 12 (*Cxcl12*; dRnoCPE5149237), integrin 1 beta (*Itgb1*; dRnoCPE5168303), interleukin 1 beta (*Ilb*; dRnoCPE5171241), proliferation marker Ki-67 (*Mki67*; dRnoCPE5167738), transforming growth factor beta (*Tgfb*; dRnoCPE5176359), vascular endothelial growth factor (*Vegf*; dRnoCPE5146882). The peptidyl–prolyl cis–trans isomerase H (*Ppih*; dRnoCPE5177930) gene was used as a reference.

### 2.9. Statistical Analysis

All data were evaluated for normality using the Shapiro–Wilk test in GraphPad Prism 9 (GraphPad Prism 9 software, Boston, MA, USA). For parametric data, Welch’s *t*-test was employed for comparing two groups. When comparing more than two normally distributed groups, one-way ANOVA with Tukey’s post hoc corrections was applied. For non-parametric data, the Mann–Whitney U-test was used for two-group comparisons, and the Kruskal–Wallis test with Dunn’s post hoc test was utilized for multiple group comparisons. A *p*-value < 0.05 was considered statistically significant.

## 3. Results

### 3.1. Confirmation of Biomaterial Production

**Figure 3:**
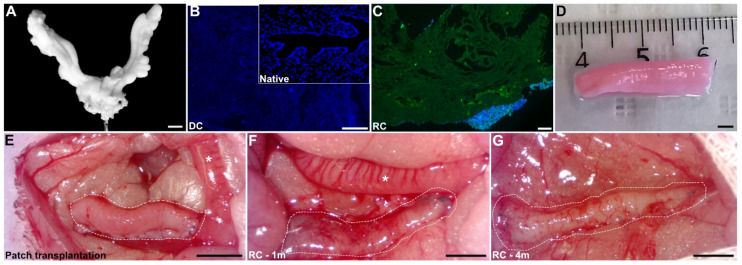
Decellularization of the rat uterus was confirmed by the white aspect of the organ (**A**) and by cell nuclei removal by DAPI staining before and after decellularization (**B**). Recellularization using GPF-labeled rat bone marrow derived mesenchymal stem cells and was assessed by immunofluorescence (anti-GFP ab290 from Abcam, 1:100 + DAPI stain) (**C**). The decellularized uterus was cut into 2 × 1 cm patches (**D**) for further transplantation into rats (**E**). Tissue regeneration was evaluated one month and four months post-transplantation (**F**,**G**). The graft is marked as a white dotted line, and the asterisks mark the native uterine horn. Scale bars: (**A**,**D**–**G**) = 500 µm; (**B**,**C**) = 75 µm.

Our previously established decellularization protocol for rat uterine tissue was confirmed, based on the absence of intracellular content and the preservation of connective and extracellular tissue though DAPI staining (Figure 3A,B). Immunohistochemistry on recellularized uterine scaffolds showed that the cells localized primarily to the superficial layers of the decellularized tissue, particularly covering the external surface area of the scaffold (Figure 3C,D).

### 3.2. Observations During Graft Retrieval

All grafts retained their shape during the first month, showing no signs of lumen collapse. There were no obvious discrepancies in appearance between the acellular and recellularized grafts. However, some animals developed graft adhesions to the adjacent omentum and intestinal tissues, and a few grafts exhibited stenosis at the anastomosis site, leading to fluid accumulation proximal to the lesion.

By four months, some grafts had collapsed leading to a reduced luminal diameter compared to normal tissue. Several grafts also appeared to have lost their structural integrity and size. Tissue adhesions to surrounding tissues were also more prominent compared to the one-month time point. However, no obvious macroscopic differences were noted between the study groups (Figure 3E–G).

### 3.3. Uterus Tissue Regeneration Evaluation

**Figure 4:**
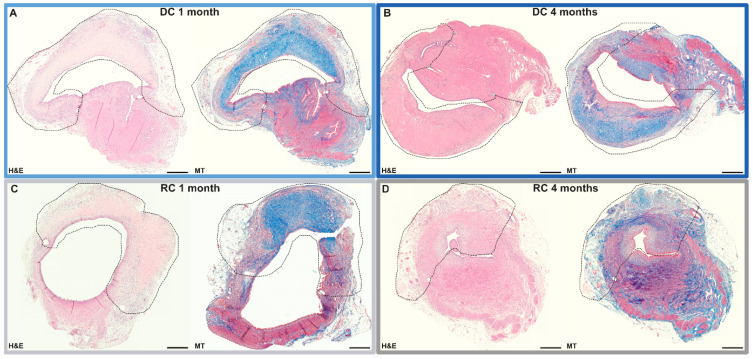
Histological assessment with hematoxylin and eosin staining (H&E) and Masson’s trichrome staining (MT) on acellular grafts (DC) (**A**,**B**) and grafts recellularized with bone marrow-derived mesenchymal stem cells (RC) (**C**,**D**), one month (1 m) and four months (4 m).

Each specimen was thoroughly assessed microscopically with particular focus on tissue regeneration. After one month, the grafts showed a wide range of regeneration level (Figure 4; Table 1), where n = 2 were graded “Partial”, n = 1 as “Moderate”, and n = 3 as “Substantial” for the DC group. The outcomes for the RC group after one month were n = 3 as “Minor”, n = 2 as “Partial”, and n = 0 as “Moderate”, and n = 1 as “Substantial”. 

Four months after transplantation, the DC grafts had n = 2 as “Minor”, n = 0 as “Partial”, and n = 1 as “Moderate”, and n = 2 as “Substantial”. For the RC group at 4 months post patch transplantation, n = 1 was graded “Minor”, n = 0 as “Partial”, and n = 1 as “Moderate”, and n = 2 as “Substantial”, and n = 1 as “Major”.

**Figure 5:**
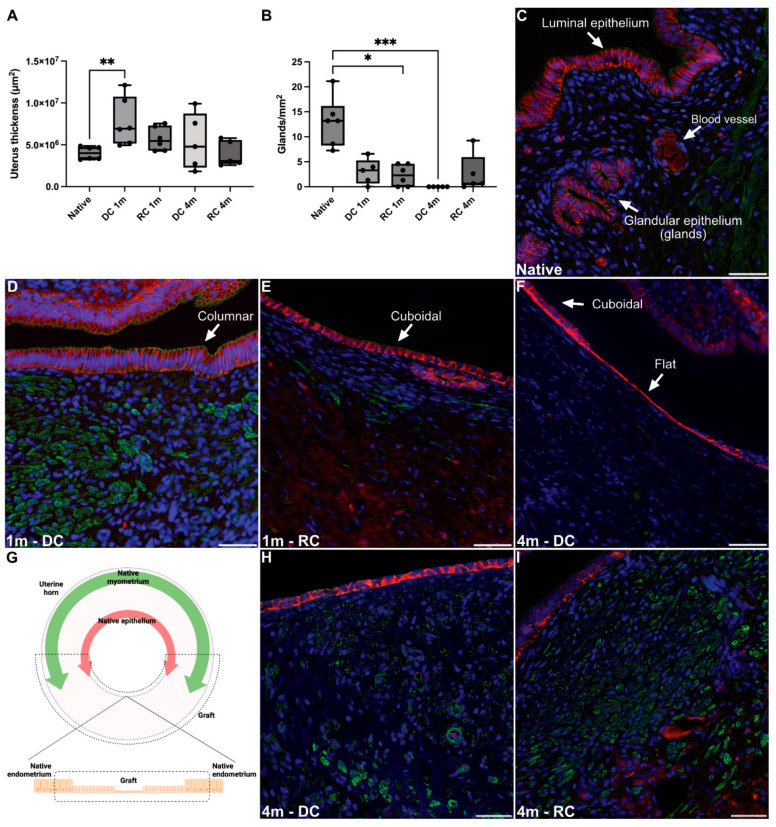
Morphological assessment of uterine tissue regeneration; evaluation of uterine thickness (**A**), number of glands (**B**,**C**), luminal epithelium morphology (**D**–**G**), myometrium regeneration (**G**–**I**). Immune labeling: (**C**–**F**,**H**,**I**), Ecad = red, aSMA = green, DAPI = blue; Scale bars; (**C**–**F**,**H**,**I**) = 50 µm. * *p* < 0.05, ** *p* < 0.01, *** *p* < 0.001.

When the cross-sectional area of the uterine grafts was measured, we detected a significant hyperplastic change in the acellular grafted scaffolds after one month. This tissue swelling had subsequently decreased after four months and was similar compared with the cross-sectional area of normal uterus tissue (Figure 5A).

**Figure 6:**
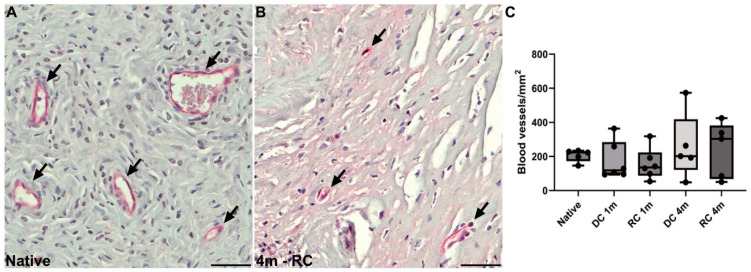
Morphological assessment of uterine tissue regeneration by blood vessel quantification. (**A**,**B**) CD31 = red, blue-black = DNA; Scale bars; (**A**,**B**) = 100 µm. Black arrows specify identified blood vessels that were quantified (**C**).

E-cadherin-stained luminal and glandular epithelium indicated that the endometrial glandular structures failed to regenerate in several grafts, particularly in the acellular grafts four months post-surgery where no glands were visible (Figure 5B,C). Interestingly, upon assessing the luminal epithelium from all grafts, we observed distinct stages of epithelial regeneration. For example, mature polarized epithelial cells were predominantly found closer to the anastomosis site of the grafts one-month post-surgery, while cells located further from the native uterine tissue exhibited an immature flat/multilayered-to-cuboidal phenotype. Recellularized grafts demonstrated a higher degree of completely regenerated polarized luminal epithelium compared to acellular grafts at the four-month time point (Figure 5D–G). Similarly, αSMA-positive myometrial cells appeared to regenerate from the native surrounding uterine tissue and migrate inwards to the more distant areas of the grafts. This progression of regeneration was more evident in the recellularized grafts and seemed to have occurred more rapidly (Figure 5H,I). No significant difference was found between any of the groups or time points when graft revascularization was assessed (Figure 6A–C).

### 3.4. Expression Analysis for Growth-Related Genes

**Figure 7:**
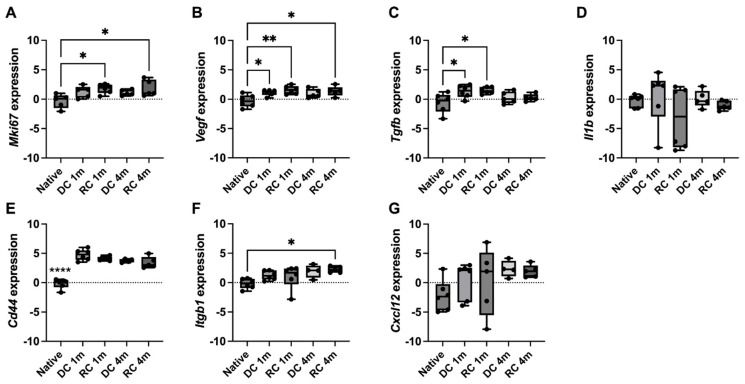
The gene expression for *Ki67* (**A**), *Vegf* (**B**), *Tgfb* (**C**), *Il1b* (**D**), *Cd44* (**E**), *Itgb1* (**F**), and *Cx*cl12 (**G**) was analyzed in cells within the scaffolds one and four months after transplantation and were compared to native uterus tissue gene expression. We confirmed an upregulation in *Vegf*, *Tgfb*, and *Cd44* in all grafts, and mesenchymal stem cells prolonged the elevated levels of *Ki67*, *Itgb1*, and *Vegf* genes. Expression plotted as Log_2_(fold change). * *p* < 0.05, ** *p* < 0.01, **** *p* < 0.0001.

The gene expression was measured from tissue samples isolated from grafted tissue; all samples showed good RNA purity parameters (median [min–max]: A260/A280 = 1.982 [1.831–2.010], A260/A280 = 2.065 [1.259–2.213]) after the extraction. There was a significant increase in the proliferation marker Ki67 (*p* = 0.0185 in RC 1 m; *p* = 0.0200 in RC 4 m) and the angiogenesis-related *Vegf* gene (*p* = 0.0031 in RC 1 m; *p* = 0.0150 in RC 4 m) in that the RC grafts for both timepoints compared with native uterine tissue (Figure 7A,B). The *Vegf* expression was also elevated in the DC grafts one month after surgery compared with native tissue (*p* = 0.0344). The *Tgfb* expression was elevated during the first month after surgery for both groups (*p* = 0.0349 in DC 1 m; *p* = 0.0205 in RC 1 m; Figure 7C), while no significant difference in expression levels was seen for *Il1b*, *Cd44* or *Cxcl12* genes. *Integrin beta 1* was significantly raised in the RC grafts at the four-month time point (*p* = 0.0164; Figure 7D–G).

## 4. Discussion

Earlier studies showed that transplanted decellularized uterine tissue with or without added cells provided regenerative support in vivo, following uterus injury in both small and large animal models [25,26,27]. However, most of these in vivo studies utilized small grafts in the rodent model (≤15 × 0.5 mm). In the present study, we used a relatively large graft (20 × 10 mm), with a size comparable to the size of an entire uterus horn of the rat. Despite presenting greater challenges for structural support, revascularization and uterus tissue regeneration following transplantation, this graft size allows us to evaluate more clinically relevant bioengineering challenges. An initial study using the same model showed that grafts recellularized with bmMSCs enhanced tissue regeneration by modulating the immune cells towards a regenerative stage [19]. The objective of our study herein was to extend these observations and conduct analysis of the histopathological events and further to investigate possible discrepancies in gene expression for some relevant growth-related proteins, in order to elucidate possible long-term regenerative mechanisms, including four months following transplantation.

The histological evaluation of the uterus tissue regeneration was based on a 5-grade scheme, modified from our previously developed scoring system for sheep uterus tissue regeneration [21]. This scoring did not generate results that proved any obvious significant benefits for grafts containing bmMSCs. However, the significant increased graft size in acellular grafts one month after transplantation suggest hyperplasia and an increased inflammation compared with the RC group. This corroborates with earlier observations with an increased graft infiltration of CD45^+^ leukocytes one month after transplantation [19]. The scoring did not generate results that proved any obvious significant benefits for grafts containing bmMSCs. However, the significant increased graft size in acellular grafts one month after transplantation suggests hyperplasia and an increased inflammation in DC compared with the RC group. This corroborates with earlier observations of an increased graft infiltration of CD45^+^ leukocytes one month after transplantation [8]. Collectively, these results suggest that it should not be necessary to use fully reconstructed bioengineered uterine grafts, but one may consider the endogenous self-repair mechanisms that take place in situ. Yet, it becomes clear that these events can therapeutically be potentiated with bmMSCs, or possibly with their extracellular vesicle products, or other chemotactic factors yet to be identified [26,27,28]. For example, a recent xenograft study used extracellular vesicles derived from mouse adipose-derived MSCs together with decellularized pig skin to stimulate rat endometrial regeneration after luminal administration in vivo [29].

We did not notice any difference in the number of blood vessels between the studied groups and native tissue, suggesting an effective bmMSC-independent revascularization already one month after transplantation. However, we observed that cells within the scaffolds still had a potentiated gene expression of *Vegf* during the first month, indicating that the newly formed capillaries grow in volume rather than in numbers. The overexpression of *Vegf* had ceased in the acellular grafts but remained higher in the recellularized grafts at the four months time point. Earlier studies have shown that decellularized uterus tissue possess bioactive cues even after harsh chemical treatment, stimulating angiogenesis in the chick embryo chorioallantoic membrane assay [1,15,30,31]. Hence, increased *Vegf* expression might also partly be induced by growth cues within the scaffold, and not only by the bmMSCs during the first month after engraftment. Interestingly, the expression of VEGFs downstream signaling pathway protein CD44 was also elevated in both experimental groups and both timepoints. CD44 is a cell surface glycoprotein that can act as a co-receptor for various growth factors, including VEGF [32]. It has been shown to facilitate VEGF-mediated signaling pathways and promote cell proliferation, migration, and survival. Another VEGF-induced signaling pathway protein is ITGB1 [33]. This gene was overexpressed in the RC group four months after surgery. Increased *Itgb1* expression has been found to accelerate muscle regeneration in mice by an enhanced ITGB1 binding activity to laminin stimulated myogenesis mediated by the activation of the focal adhesion kinase (FAK)-dependent extracellular signal-regulated kinase (ERK) and activated protein kinase (AKT) signaling axes, respectively [34]. Hence, the elevated *Itgb1* expression in the recellularized grafts four months after surgery may be responsible for the better organized myometrium layer in this group. The significant elevation of the proliferation-related *Ki67* gene expression in RC scaffolds provides additional evidence to this theory and may explain the more rapid regenerative progress seen in grafts recellularized with bmMSCs.

VEGF signaling also cross-talks with the TGFB pathways [35]. When activated, TGFB plays an important role in regulating inflammatory progression and wound healing, but also mediates pathways for cell differentiation, proliferation, and migration [36]. Cells within all grafts experienced an elevated *Tgfb* expression during the first month following surgery, and together with the noted *Cd44* and the *Itgb1* expression elevation, these activated pathways could explain the mechanism behind the luminal epithelialization and myometrial reformation observed. This might also explain the rapid revascularization of the grafts, since these pathways play an important role in neovascularization and vascular smooth muscle cell proliferation [37]. However, TGFB is also involved in the epithelial–mesenchymal transition (EMT) and can be an activator for fibrotic disease, including uterus scarring and intrauterine adhesions after endometrial injury [38]. MSCs-derived exosomes were shown to decrease EMT pathway activation by reducing the TGFB and improve endometrial regeneration [28]. The increased *Tgfb* expression we detected in the first month after surgery may thus reflect an improved epithelialization but may to some degree negatively affect the endometrial regeneration. Indeed, the glandular endometrial layer failed to regenerate adequality in our study. Future studies will include recellularizing the scaffolds with endometrial stem-like phenotype cells that showed promising in other studies [39]. Combination therapy together with decellularized tissue, MSCs and/or exosomes may be required to restore all tissue layers of the uterus and restore fertility.

More extensive signaling pathway analysis that derives the regenerative mechanisms should be included in future studies. For example, it has been shown that the uterus tissue repair following decellularized tissue transplantation was estrogen and progesterone independent, but signal transducer and activator of transcription 3 (STAT3) and leukemia inhibitory factor (LIF) dependent [8,40,41,42]. Hence, a limitation of our study is that these pathways were not further assessed in our experimental setting. Future studies should include multi-omics analysis to thoroughly assess the pathways involved in uterine repair [42,43,44] to decipher new potential therapeutic targets. Furthermore, utilizing large grafts in our animal models may require further scaffold modifications, e.g., we noticed that the scaffold in some animals appeared to have degraded faster than the ongoing regeneration progress, which may have led to an insufficient support. Hence, scaffold modifications to delay the degradation time should also be considered in future studies, e.g., through utilizing crosslinking strategies using genipin or procyanidins [9], or implementing a hybrid composite sandwich layer of polyurethane, poly (lactic-co-glycolide), or collagen together with the decellularized tissue [45,46,47].

## 5. Conclusions

We found that a significantly large uterine injury could be regenerated following decellularized tissue transplantation, independently of bmMSC repopulation. However, when this cell type was added to the scaffold, a more rapid activation of endogenous repair mechanisms occurred. We identified elevated expression of *Vegf*, *Tgfb*, and *Cd44* in cells within the graft. Recellularized grafts also potentiated the *Ki67* and *Itgb1* expression. These activated genes seem involved in the more rapid recolonization and regeneration of the luminal epithelial layer and the myometrial layer in the recellularized grafts.

## Figures and Tables

**Table 1:** Regeneration was scored by two individual lab members blinded to the study groups, adhering to histopathologic scoring guidelines demonstrated elsewhere [21,22].

Scoring of Uterus Tissue Regeneration						
Group	Minor (0)	Partial (1)	Moderate (2)	Substantial (3)	Major (4)	Average Score
DC 1 month	0	2	1	3	0	2.2
RC 1 month	3	2	0	1	0	1.5
DC 4 months	2	0	1	2	0	2
RC 4 months	1	0	1	2	1	2.4

(0) Minor; Degraded and non-uniform/irregular graft structure/morphology. Tissue layers (endometrium, myometrium) are undistinguishable. No visible glands in the endometrium. (1) Partial; Uniform graft structure/morphology. Tissue layers (endometrium, myometrium) are undistinguishable. No visible glands in the endometrium. (2) Moderate; Uniform graft structure/morphology. Endometrium is distinguishable but without visible glands. The myometrium is undistinguishable, or not completely structured. (3) Substantial; Uniform graft structure/morphology. The myometrium is undistinguishable, or not completely structured. Alternatively, distinguishable myometrium but no visible glands in the endometrium. (4) Major; Uniform graft structure/morphology. Endometrium is distinguishable with visible glands. The myometrium is distinguishable and completely structured, comparable to normal tissue.

## Data Availability

All data presented herein are available from the corresponding author on reasonable request.
